# Development of Eco-Friendly Biocomposite Films Based on *Opuntia ficus-indica* Cladodes Powder Blended with Gum Arabic and Xanthan Envisaging Food Packaging Applications

**DOI:** 10.3390/foods13010078

**Published:** 2023-12-25

**Authors:** Malha Oudir, Zohra Ait Mesbah, Djahida Lerari, Nadia Issad, Djamel Djenane

**Affiliations:** 1 Higher School of Food Science and Agri-Food Industry, ESSAIA, Avenue Ahmed Hamidouche Route de Beaulieu, El Harrach, Alger 16200, Algeria; oudir@essaia.dz (M.O.); n_issad@essaia.dz (N.I.); 2 Fundamental and Applied Physics Laboratory, FUNDAPL, Faculty of Science, University of Blida 1, P.O. Box 270, Route de Soumâa, Blida 09000, Algeria; haneddz@yahoo.fr; 3 Center for Scientific and Technical Research in Physical and Chemical Analysis, CRAPC, Zone Industrielle Bou-Ismaïl, P.O. Box 384, Tipaza 42004, Algeria; lerari_zinai@yahoo.fr; 4 Laboratory of Food Quality and Food Safety, Mouloud Mammeri University, P.O. Box 17, Tizi Ouzou 15000, Algeria

**Keywords:** *Opuntia ficus-indica*, cladodes powder, gums, blend films, eco-friendly, food packaging

## Abstract

Currently, food packaging is facing a critical transition period and a major challenge: it must preserve the food products’ quality and, at the same time, it must meet the current requirements of the circular economy and the fundamental principles of packaging materials eco-design. Our research presents the development of eco-friendly packaging films based on *Opuntia ficus-indica* cladodes (OFIC) as renewable resources. OFIC powder (OFICP)-agar, OFICP–agar-gum arabic (GA), and OFICP–agar-xanthan (XG) blend films were eco-friendlily prepared by a solution casting method. The films’ properties were investigated by scanning electron microscope (SEM), Fourier transform infrared spectroscopy (FTIR), X-ray diffraction (X-RD), and differential scanning calorimeter (DSC). Water solubility and moisture content were also determined. Morphology, thickness, molecular interactions, miscibility, crystallinity, and thermal properties, were affected by adjusting the gums (GA and XG) content and glycerol in the blend films. Moisture content increased with increasing glycerol and XG content, and when 1.5 g of GA was added. Water solubility decreased when glycerol was added at 50% and increased with increasing GA and XG content. FTIR and XRD confirmed strong intermolecular interactions between the different blend film compounds, which were reflected in the shifting, appearance, and disappearance of FTIR bands and XRD peaks, indicating excellent miscibility. DSC results revealed a glass transition temperature (T_g_) below room temperature for all prepared blend films, indicating that they are flexible and soft at room temperature. The results corroborated that the addition of glycerol at 30% and the GA to the OFICP increased the stability of the film, making it ideal for different food packaging applications.

## 1. Introduction

Food packaging is essential for preserving the quality of foods products; it is a barrier to different factors of food deterioration such as oxygen, light, and moisture. Further, it provides physical protection, prevents post-processing contamination, and extends shelf life of foods products [1,2,3]. Paper, metal, and plastic are the most materials used for food packaging. Nevertheless, plastic is the material most widely used in the food industry due to its various advantages, such as excellent physicochemical and mechanical properties, high processability, flexibility, stability, and excellent thermal resistance [4,5]. In fact, three hundred and ninety-one (391) million metric tons of plastic were produced worldwide in 2021, and 40% of these are dedicated to packaging manufacturing [6]. However, the impact of plastic on the environment is devastating,; only 9% of the produced plastic is recycled, and the remainder is disposed of in uncontrolled dumps or is released into the environment as waste, thus causing soil, water, and air pollution, ecological imbalance, climate change, and endocrine disruption, even at very-low doses [6,7].

Therefore, tackling plastic is becoming a primary environmental, scientific, industrial, and societal concern. Unfortunately, this is not easy; indeed, food packaging is currently undergoing a crucial transition period and is facing a major challenge. It needs to ensure the preservation of food product quality while fulfilling the current requirements of the circular economy and new complex regulations for ensuring the safety of packaging materials, in particular, the potential risk of migration of additives known to be endocrine disruptors such as bisphenol A and phthalates [8].

The food industry has experienced great development in recent years which has contributed to the intensified creation of innovative solutions in the packaging industry [9,10,11,12]. However, the packaging industry faces certain challenges when preparing potential food packaging materials. Increasingly, packaging manufacturers must also be attentive to their environmental impact throughout supply chains. Today, a number of solutions exist to reduce the carbon footprint of food packaging (recyclable and bio-sourced materials, etc.). The materials must also be economically profitable, appropriate for widespread application, and ensure safety for human health. Biopolymers derived from natural resources have recently attracted great attention in food packaging materials as an alternative to non-biodegradable plastic packaging due to their abundance, compatible mixtures of different components that can easily be eco-friendly, renewable, inexpensive, and non-toxic [13,14,15,16,17].

*Opuntia ficus-indica* (OFI) cladodes (OFICs) are one of the trendy renewable resources that are attracting currently growing interest in the food packaging field due to their richness in carbohydrates, including pectin, fiber, mucilage, lignin, cellulose, and hemicellulose, as well as their wide availability [18]. In fact, the *Opuntia ficus-indica*, commonly known as cactus or nopal, is cultivated all over the world, mainly in the western part of the Mediterranean basin (southern Portugal, Spain) and in North Africa (Algeria, Morocco and Tunisia) [19]. The OFI plantation covers 100,000 hectares through Algerian territory. Of this, 60% is situated in the Sidi-Fredj region (southeast of the Wilaya of Souk Ahras) [20]. It is estimated that one hectare of OFI produces 100 tons of cladodes per year [21,22].

As mentioned above, OFICs have recently attracted a great deal of attention in food packaging applications. Many studies have employed their mucilage for film development [23,24,25,26,27,28,29]. Gheribi et al. [26] reported that cladodes’ mucilage exhibited good film-forming properties when plasticized and thus can form film for food packaging. The different applications of OFIC mucilage in food packaging were reviewed by Gheribi and Khwaldia [28] and de Andrade et al. [23].

Due to their antimicrobial and antioxidant activity and their richness in bioactive compounds, OFIC mucilage is investigated as an interesting candidate for developing bioactive packaging films and coatings, which are applied to extend the shelf life of highly perishable foods such as meats and fruits [3,22,28,30,31].

However, in addition to low yield, the mucilage extraction from OFICs has traditionally involved organic solvents that are derived from non-renewable resources and are suspected of being harmful to humans and the environment [25,26,29]. This practice does not conform to the fundamental eco-design principles of packaging materials, contrary to the use of whole powder without any organic solvents which is part of an environmentally friendly approach. Nevertheless, there is no study report on the use of OFIC powder (OFICP) as a single material, without any addition, for film development. The few studies that have been carried out with OFICP have used it as either a filler and as a reinforcing agent in starch films [32] or blended with other biopolymers to prepare biocomposites [32,33].

Blending two or more biopolymers allows for combining of their properties and improves the properties of the resulting blend film by intermolecular interactions between the different biopolymers favored by their natural thermodynamic compatibility [34,35]. In this context, the addition of natural gums, such as agar, XG, and GA, into the blend biomaterials of food packaging has currently received further attention due to their many functional properties. Particularly, their biodegradability, low cost, film-forming properties, as well as the presence of several hydroxyl groups (–OH) in their chemical structure that interact with the other blend components through hydrogen bonding has thus resulted in appearance improvement, changes in solubility, and structural and thermal property modifications in the resulting blend film [13,35,36,37,38,39,40].

Although the scientific literature on film prepared from the combination of two or more biopolymers is currently extensive [35], as far as we know, there has only been one study conducted on investigating the combination of OFICP and agar, as gum, for preparing OFICP–agar bend film with different concentrations of glycerol (as plasticizer). The obtained OFICP–agar blend film had a moderate mechanical performance, thermal stability, and water vapor transmission rate [32].

However, the thermal properties and intermolecular interactions between OFICP and agar were not reported in the mentioned study. Furthermore, to the best of our knowledge, the OFICP–agar-XG and OFICP–agar-GA ternary blend films have also not been reported to date. It is therefore necessary to investigate the effects of this combination on the behavior of the blend films to determine its suitability for food applications. In this context, the main objective of the present study was to investigate the effect of glycerol, GA, and XG addition on the appearance, thickness, morphology, moisture content, water solubility, crystallinity, intermolecular interactions, and thermal properties of the OFICP–agar blend film.

## 2. Materials and Methods

### 2.1. Materials

A total of 10 kg of OFIC were harvested during 2021 in the region of Haizer (latitude of 36,397; longitude of 399,917, 36°23′49″ North, 3°59′57″ East; altitude: 609 m), with an annual average temperature of 16 °C and precipitations of 650 mm/year, located at 9 km from Bouira in northern Algeria. The harvest was performed in cardboard bags.

**Figure 1:**
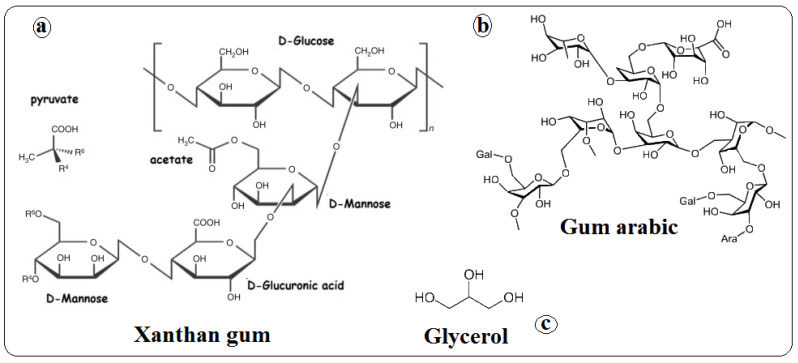
Chemical structure of (**a**) xanthan (XG), (**b**) gum arabic (GA), and (**c**) glycerol (G).

The cladodes were selected according to size: 30–35 cm in length and 10 cm in width. Gum arabic, xanthan (food grade), agar, and glycerol (analytical grade) (Figure 1) were purchased from Sigma-Aldrich (≥99.5% purity) (St. Louis, MO, USA).

### 2.2. Preparation of Opuntia ficus-indica Cladodes Powder (OFICP)

The OFICP was prepared according to Makhloufi et al. [32], with minor modifications. The spines were manually removed, and then the cladodes were washed with tap water, rinsed with distilled water, disinfected with sodium hypochlorite solution 200 mg/L (Sigma Aldrich^®^-Química, Madrid, Spain), and dried with paper towels, cut into small cubes, and dehydrated in a ventilated oven (J.P. Selecta, Barcelona, Spain) at 60 °C for 24 h. The dehydrated pads were ground and sieved through 500, 200, 100, and finally, 45 µm mesh sieves (Retsch, Haan, Germany) to obtain a green powder with fine granulometry Ø < 45 µm. The obtained OFICP was stocked in an airtight glass bottle away from light and moisture for further use.

### 2.3. Blend Films Preparation

The solvent casting method was used to prepare the blend films with three different formulations based on the method described by Makhloufi et al. [32], with minor modifications. The first formulation was prepared with a constant and equal amount of OFICP (2 g) and agar (2 g), 1:1 ratio, with varying concentrations of glycerol (0%, 30%, 40%, and 50%, *w*/*w*). The second formulation was prepared at 30% glycerol with 2 g of OFICP and 2 g of a blend of agar and GA prepared at the following ratios (agar: GA); (2:0), (1.7:0.3), (1.5:0.5), (1:1), and (0.5:1.5). The third formulation was also prepared at 30% glycerol with 2 g of OFICP and 2 g of a blend of agar and XG prepared at the following ratios (agar: XG): (2:0), (1.7:0.3), (1.5:0.5), and (1:1). The different prepared film formulations are listed in Table 1.

**Figure 2:**
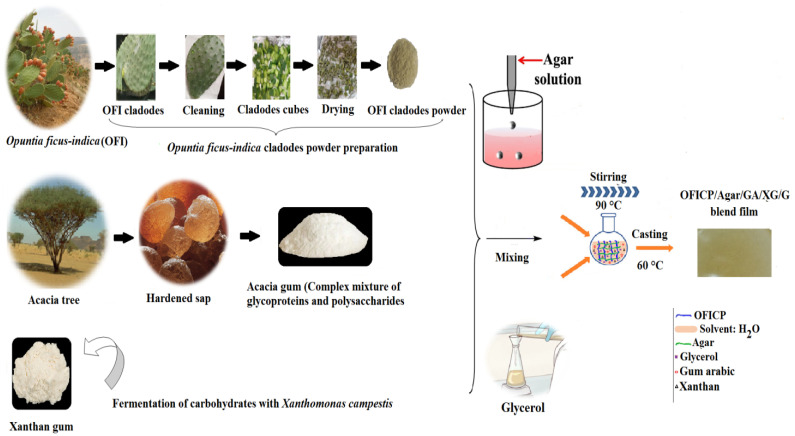
Preparation scheme of the agar/OFICP-GA and agar/OFICP-XG ternary blend films.

The film-forming solutions were prepared according to Makhloufi et al. [32]. Firstly, 2 g of OFICP was dissolved in 100 mL of distilled water and stirred for 30 min at 90 °C. After that, the blends of agar, GA, XG, and glycerol were added as plasticizer at the previously specified amounts. The mixture was constantly stirred at 90 °C for 15 min to obtain a film-forming suspension. Then, 10 mL of each suspension was cast on an 80 mm diameter Petri dish and dried in an oven at 40 °C for 24 h. Once dried, the films were peeled off and stored in a desiccator with silica gel at room temperature. The manufacturing process of the ternary blend films is shown in Figure 2.

### 2.4. Visual Control

All the obtained blend films were visually inspected to determine the appearance, color, texture, resistance, and presence of any impurities.

### 2.5. Film Thickness Measurement

The thickness of the obtained blend films was measured using Mitutoyo coolant-proof digimatic micrometers MDC-25PX at an accuracy of ± 0.001 mm (Mitutoyo Corporation, Tokyo, Japan) at random different points around the film. In total, thirty (30) measurements per formulation were performed, with three (3) films per formulation at ten (10) different positions per film. Mean values ± SD were determined.

### 2.6. Scanning Electron Microscope (SEM)

The morphology of all samples (starting materials and prepared films) was analyzed by a scanning electron microscope (SEM, Quanta 650, Rock Hill, SC, USA) operated at 10 kV. Each sample was put on a steel plate and coated with a carbon film before analysis.

### 2.7. Moisture Content

The moisture content of the prepared blend films was determined by a gravimetric method according to Gheribi et al. [26]. Samples of 2 cm × 2 cm were weighed (m*i*), oven-dried for 24 h at 90 °C, and then reweighed (m*f*). The moisture content was calculated as the percentage of dried weight loss as follows:

$$\mathrm{Moisture}\;\mathrm{Content}\;(\%)=\frac{mi-mf}{mi}\times 100$$

Results were obtained from at least 3 independent experiments carried out on different working days. Mean values ± SD were determined.

### 2.8. Water Solubility

The water solubility of the prepared blend films was determined according to Gheribi et al. [28]. Film samples of 2 cm × 2 cm were oven-dried at 90 °C for 24 h, cooled to room temperature in a desiccator, weighed (m*i*), and then immersed in 50 mL of distilled water at room temperature for 30 min. The undissolved pieces were oven-dried at 90 °C for 24 h and then weighed (m*f*) after cooling to room temperature. The film’s water solubility was determined according to:

$$\mathrm{Water}\;\mathrm{solubility}\;(\%)=\frac{mi-mf}{mi}\times 100$$

Results were obtained from at least 3 independent experiments carried out on different working days. Mean values ± SD were determined.

### 2.9. Fourier Transform Infrared (FTIR) Spectroscopy

FTIR analysis was used to determine the functional groups of the starting materials and intermolecular interactions that occurred between them in the blend films. The FTIR spectra of the starting materials and the prepared blend films were recorded using a Fourier transform infrared spectrometer (FTIR-8900, Shimadzu, Japan) in the wavenumber range of 4000–400 cm^−1^ with a resolution of 4 cm^−1^ and 32 accumulated scans.

### 2.10. X-ray Diffraction (XRD)

X-ray diffraction analysis was used to characterize the structure of the starting materials and their miscibility in the obtained blend films, as well as to determine the crystallinity index. The X-ray diffraction patterns of the starting materials (OFICP, agar, GA, and XG) and the prepared blend films were measured by an X-ray diffractometer (Panalytical Empyrean, United Kingdom), with Cu-K alpha radiation (λ = 1.54060 Å) under the voltage of 45 kV and 40 mA. The diffractograms were recorded in the 2θ range between 10° and 60° with a step size of 0.0130. OriginPro 2022 software was used to plot the graphs. The crystallinity index (%) was calculated as the ratio of the crystalline area of the individual peaks and the total area of the total diffractogram [41,42]:

$$\mathrm{Crystallinity}\;\mathrm{index}\;(\%)=\frac{\mathrm{Crystalline}\;\mathrm{peaks}\;\mathrm{area}}{\mathrm{Total}\;\mathrm{area}(\text{crystalline}+\text{amorphous})}\times 100$$

### 2.11. Differential Scanning Calorimeter (DSC)

Thermal properties for the prepared blend films were determined using a differential scanning calorimeter (Setaram DSC 131 Evo, ENTEC, Sarl Constantine, Algeria). About 5 mg of the samples were sealed in aluminum pans, and measurements were performed with three heating cycles separated by a cooling cycle. Thermal cycles were conducted from −70 to 100 °C with a heating rate of 10 °C/min under nitrogen (N_2_) flow rate of 40 cm^3^/min. The glass transition temperature (T_g_ °C) and the heat capacity change (∆C p) were obtained from the thermogram.

### 2.12. Statistical Analysis

The data were analyzed with one-way analysis of variance (ANOVA) using SPSS software (version 21). The results are presented as mean ± standard deviation (SD) of the triplicate measurements for moisture content, water solubility, and thickness. Tukey’s test was used to determine significant differences between the blend film properties results at *p* < 0.05.

## 3. Results and Discussion

### 3.1. Film Appearance

**Figure 3:**
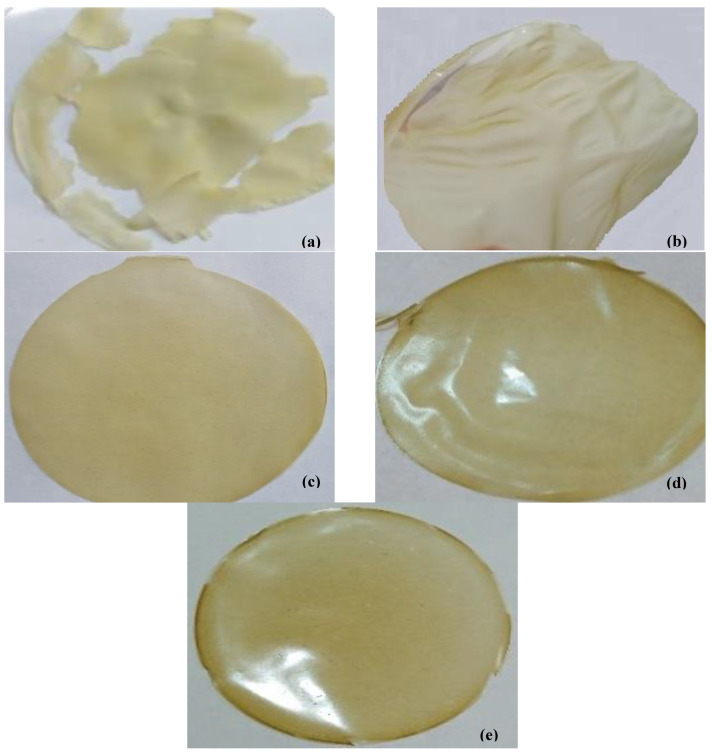
Appearance of the prepared blend films (**a**) OFICP without any addition: control, (**b**) OFICP–agar (F1-0%G), (**c**) OFICP–agar-glycerol 40% (F1-40%G), (**d**) OFICP–agar-glycerol 30%-GA0.5g (F2-0.5GA), (**e**) OFICP–agar-glycerol 30%-XG 0.5g (F3-0.5XG).

Figure 3 shows the visual appearance of the prepared blend films with the different formulations. Initially, an OFICP-based film without any addition was prepared (control). A crumbled green-color film was obtained with a granular aspect (Figure 3a). This result suggests that the OFICP has poor film-forming properties and cannot form a seamless and smooth film, which could be attributed to the OFICP’s heterogeneity, as confirmed by the FTIR results. However, the addition of agar at a 1:1 ratio into OFICP-based film without added glycerol (F1-0%G) relatively improved the film appearance, which was homogeneous, smooth, and without cracks or air bubbles, However, its appearance was very glassy and brittle (Figure 3b). This improvement may be due to the excellent gelling power of agar. The film (F1-40%G) became more flexible and manual stretch-resistant after adding glycerol at 40% (Figure 3c). Similar observations were reported by Makhloufi et al. [32], De Farias et al. [43], and Priyadarshi et al. [44] for OFICP-based film and other biopolymers with different concentrations of glycerol. At high amounts glycerol interacts with polymers through van der Waals forces, hydrogen bonding, etc. The addition of GA at 0.5 g into the formulation further enhanced the film’s appearance (F2-0.5GA), which appeared more compact, more homogeneous, smoother, more resistant, and bright, without cracks or air bubbles (Figure 3d), contrary to the film that contained XG, which displayed small air bubbles (Figure 3e). The formed film with 1 g of XG (F3-1XG) was sticky and difficult to handle and remove from the Petri dish.

### 3.2. Film Thickness

Thickness is one of the most important parameters of packaging films, impacting physical properties such as handling, barriers, and mechanical properties [45,46,47]. Our results found that the higher the glycerol content in the formulation (from 0 to 50%) was, the thicker the resulting biocomposite blend films were, as shown in Table 2. Indeed, the addition of glycerol at varying concentrations to OFICP–agar blend formulations increased the film thickness from 0.056 to 0.095 mm (F1-0%G–F1-50%G), with a significant difference (*p* < 0.05) compared to the control film without glycerol (F1-0%G).

In contrast, the addition of gums only at low contents has a significant effect on the thickness. Indeed, gum arabic and xanthan addition at the low contents of 0.3 and 0.5 g (F2-0.3GA, F2-0.5GA, F3-0.3XG, F3-0.5XG) formed thicker ternary blend films with a thickness of 0.123, 0.124, 0.105, 0.101 mm, respectively, with significant difference (*p* < 0.05) compared to the control film without gum arabic and xanthan (0.069 mm); meanwhile, at the high contents of gum arabic (F2-1GA and F2-1.5GA), thinner ternary blend films were obtained with a thickness of 0.098 and 0.090 mm, respectively, with an insignificant difference (*p* < 0.05) compared to the control film without gum arabic (0.069 mm).

Similar results were reported by Jouki et al. [48] and Makhloufi et al. [32] for cress seed carbohydrate gum edible film and OFICP-based film plasticized with glycerol, as also observed by Kim et al. [49] and Rukmanikrishnan et al. [38] for XG and GA based film. These observed differences between the formed biocomposites blend films may be explained by the free volume theory of the glycerol effect, which increases the intermolecular spaces between the film chains, thus resulting in increased film thickness [40,48], as well as the increase in the solid content in the films containing GA and XG, which have the highest thickness values (0.124 and 0.105 mm, respectively). In fact, Ratna et al. [15,50] stated that biocomposite edible film thickness can be increased by adding solid materials to composite film. Similarly, Kaya et al. [51] observed that the inclusion of vegetable extract in chitosan-based film increased film thickness from 45.4 μm (control) to 99.2 μm. Furthermore, De Carli et al. [47] and Khodaei et al. [52] noted that increasing the amount of composite materials used also increases the film thickness. However, the thickness of the blend films can be also influenced by the film-forming procedure, mold surface area, and solution volume [14,47].

### 3.3. Film Morphology

**Figure 4:**
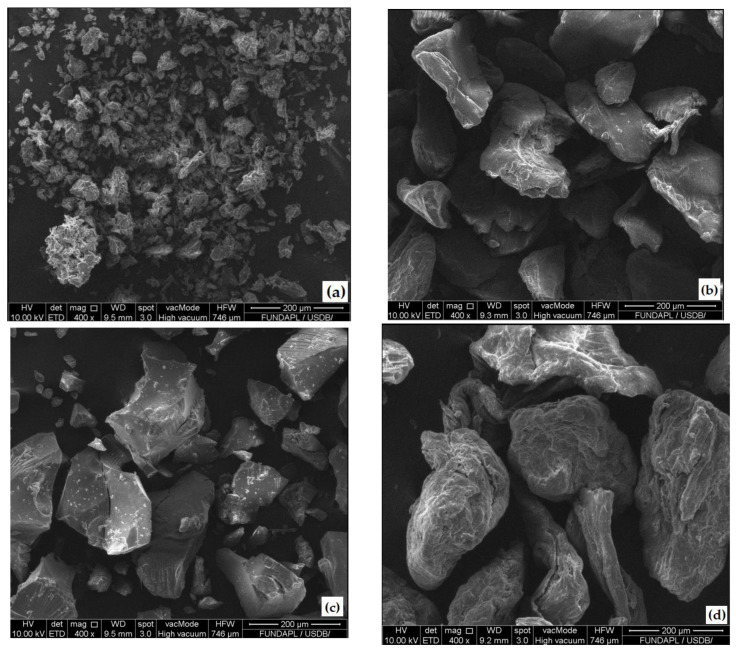

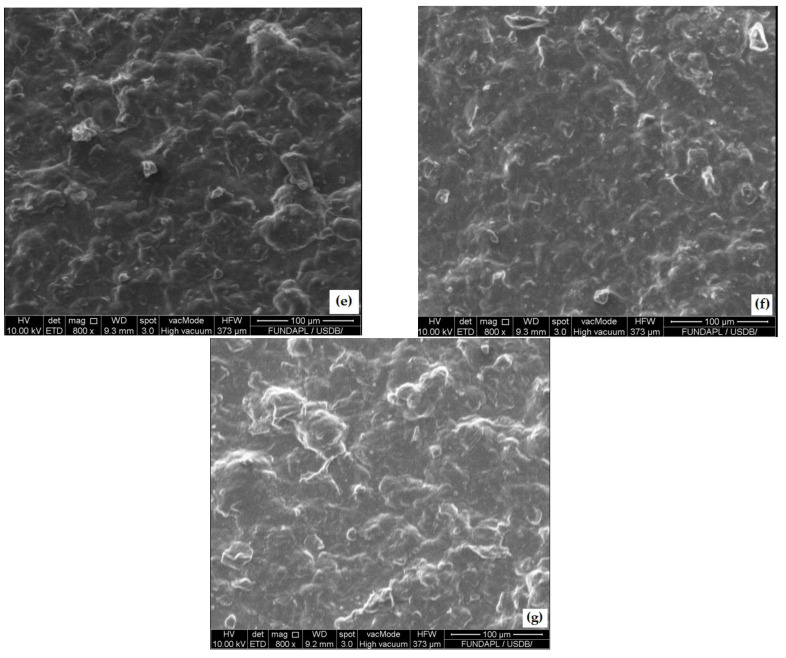
Scanning electron microscopic micrograph images of the starting materials and the prepared blend films: (**a**) OFICP, (**b**) agar, (**c**) GA, (**d**) XG, (**e**) OFICP–agar-glycerol 40% blend film (F1-40%G), (**f**) OFICP–agar-glycerol 30%-GA 1.5 g (F2-1.5GA), (**g**) OFICP–agar-glycerol 30%-XG 0.5 g (F3-0.5XG).

Figure 4 shows the SEM micrographs of OFICP, agar, XG, GA powders, and the surface of the different prepared blend films.

The SEM micrograph of OFICP (Figure 4a) showed aggregates and small particles with irregular shapes and more or less homogeneous sizes ranging from 19 to 50 µm. This may be explained by the fine granulometry of OFICP: < 50 µm. A similar micrograph was reported by Makhloufi et al. [32] for OFICP. De Farias et al. [43] indicated that powder granulometry may affect OFICP particle size.

The SEM micrograph of the powders agar (Figure 4b), GA (Figure 4c), and XG (Figure 4d) showed particles with irregular shapes and sizes ranging from 16 to 208 µm for GA, from 94 µm to 241 µm for agar, and from 278 to 440 µm for XG. Similar observations were reported by Makhloufi et al. [29]. These authors indicated that the particle size may be affected by the preparation methods of gums.

Regarding the different prepared blend films, the SEM micrographs showed a rough surface with micron particles and aggregates with irregular shapes for all blend films (Figure 4e–g). Similar results were reported by De Farias et al. [43] and Makhloufi et al. [32] for OFICP-based films; these authors suggested that this is due to the presence of fibers in OFICP. The SEM micrograph of the film containing XG (F3-0.5XG) showed an increasingly rougher surface, and as illustrated in Figure 4g, this may be due to the possible formation of XG agglomerates within the film.

### 3.4. Moisture Content and Water Solubility

**Figure 5:**
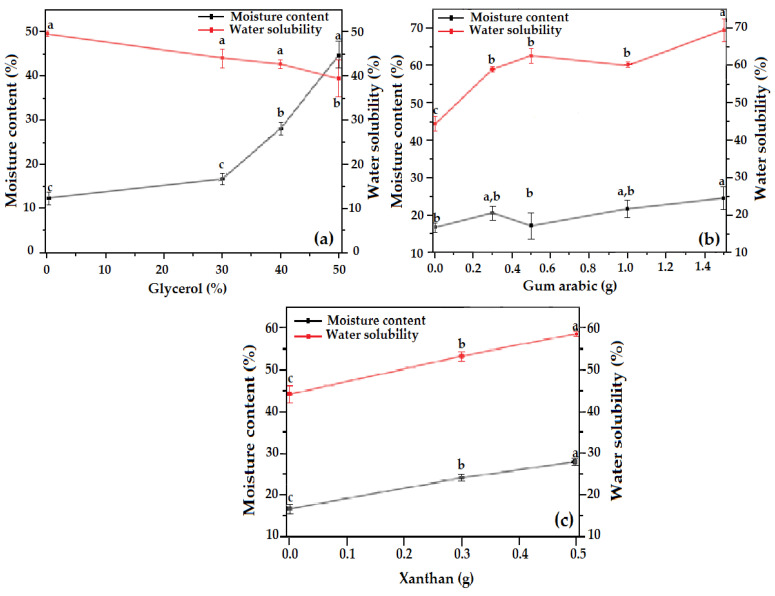
Moisture content and water solubility of (**a**) OFICP–agar blend film with different concentrations of glycerol (F1-0%G–F1-50%G), (**b**) OFICP–agar-glycerol 30% blend film with different contents of GA (F2-0.3GA–F2-1.5GA), (**c**) OFICP–agar-glycerol 30% blend film with different contents of XG (F3-0.3XG–F3-0.5XG). Different letters (a–c) indicate significant differences (*p* < 0.05).

The moisture content and water solubility values of the OFICP–agar blend films with different contents of glycerol, GA, and XG are presented in Figure 5. A significant increase (*p* < 0.05) in the moisture content was observed when the concentration of glycerol exceeded 30% (F1-40%G and F1-50%G films) (Figure 5a). This is consistent with the results of Makhloufi et al. [32], who found that the moisture content of OFICP–agar blend films increased with increasing the glycerol concentration from 30 to 60%. Similarly, various studies have reported that increasing glycerol concentration increases the moisture content of the plasticized biomaterials [26,53]. This result may be due to the higher hygroscopicity of glycerol and its higher concentration. Therefore, adding glycerol increases the –OH in the blend films, thus increasing moisture content [54,55].

Similarly, the addition of XG significantly increased (*p* < 0.05) the moisture content of the OFICP–agar-XG ternary blend films (F3-0.3XG and F3-0.5XG films) (Figure 5c). Hazirah et al. [56] observed a similar result for the gelatin–carboxymethyl cellulose-XG blend film. These authors suggest that this is due to the presence of –OH in the XG structure increasing susceptibility to water.

On the other hand, the addition of GA did not affect (*p* < 0.05) the moisture content of OFICP–agar-GA ternary blend films (F2-0.3GA, F2-0.5GA, F2-1GA). A similar result was reported by Kim et al. [49] for starch-GA blend film and by Kang et al. [37] for GA-based film. Such results may be explained by the formation of hydrogen-bonding interactions between OFICP, GA, and agar, which reduce the number of –OH that may associate with water [37,49]. Recently, Ratna et al. [15] developed an edible biocomposite gelatin film from chicken claws and observed that the water content of the biocomposite films was not affected by the amount of glycerol and carboxymethyl cellulose but was affected by the film thickness.

The water solubility of polymer is mainly related to the number of free –OH in the polymer matrix, which allow for the film to interact with the water’s hydrogen [40]. As shown in Figure 5a, there was no significant difference (*p* < 0.05) in the water solubility of the OFICP–agar blend film with 30 and 40% glycerol (films F1-30%G and F1-40%G) compared to the control film (OFICP–agar blend film without glycerol). Contrastingly, the OFICP–agar blend film with 50% glycerol (F1-50%G) showed a significant (*p* < 0.05) decrease in water solubility. Our results are consistent with those reported by Ratna et al. [15] and Hazirah et al. [56], who found that increasing glycerol concentration decreased the water solubility of corn starch- and gelatin-based films. Such a result may be due to strong interactions between the film’s compounds (OFICP, agar, and glycerol) reducing the number of free –OH. Haq et al. [57] indicated that adding a plasticizer alters the interaction between the polymer chains, which may increase or decrease the polymer’s solubility. Moreover, water solubility depends on several factors, including crosslinking between polymer chains and the structure of the polymer network [40].

On the other hand, the OFICP–agar blend film’s water solubility significantly (*p* < 0.05) increased with the addition of the gums (*p* < 0.05) (Figure 5b,c). Approximately 59% solubility was observed for films containing XG and about 70% for films containing GA, compared to 44% for films without these gums. Arismendi et al. [58], and Kim et al. [49] reported a similar result where the addition of XG and GA increased water solubility. This result may be explained by the hydrophilic nature of GA and XG, which increase the number of hydrophilic groups in a polymer matrix, thereby increasing film solubility [39,40]. Furthermore, Kim et al. [49] suggested that when films containing gums are exposed to water, the hydrogen bonds between polymeric chains become dissociated by competition with water molecules, resulting in the films’ deformation and dissolution.

### 3.5. Chemical Composition and Molecular Interaction by FTIR

**Figure 6:**
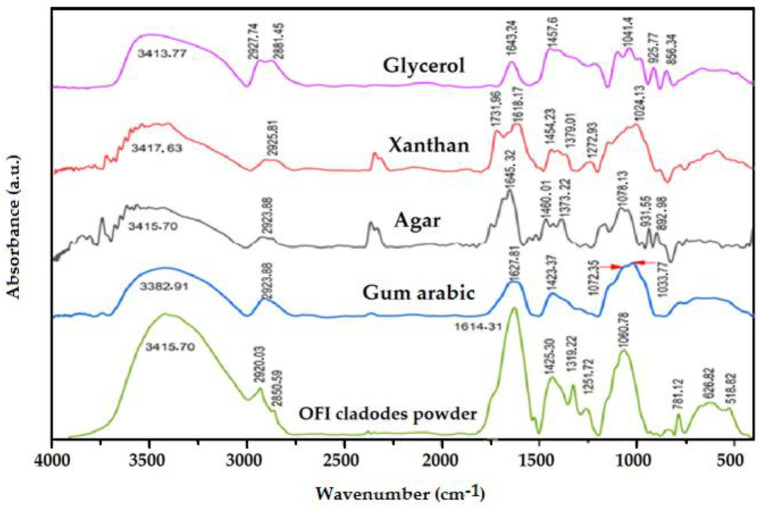
FTIR spectra of OFICP, GA, agar, XG, and glycerol.

**Figure 7:**
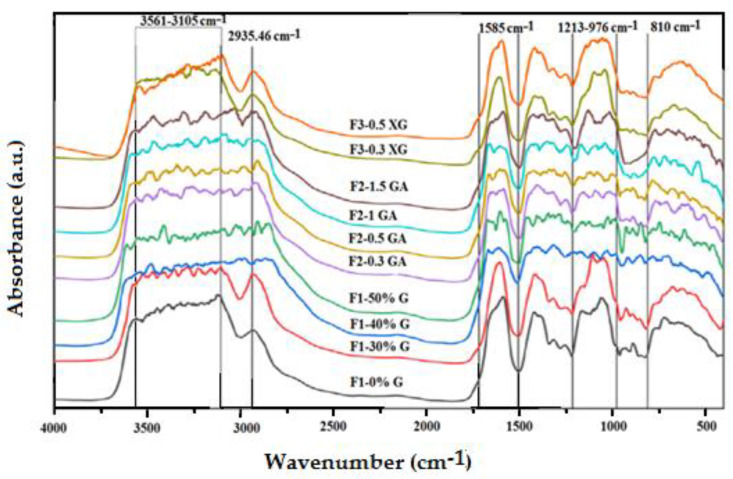
FTIR spectra of OFICP–agar blend film with different concentrations of glycerol (F1-0%G–F1-50%G) and OFICP–agar-glycerol 30% blend film with varying contents of GA (F2-0.3GA–F2-1.5GA) and XG (F3-0.3XG–F3-0.5XG).

FTIR analysis was performed to characterize the nature of the starting materials’ functional groups and the intermolecular interactions between them in the blend films. Figure 6 shows the FTIR spectra of OFICP, agar, GA, and XG, while Figure 7 shows the FTIR spectra of the manufactured blend films.

FTIR spectrum of the OFICP showed several characteristic bands appearing in two regions: 3700–2200 cm^−1^ and 1800–500 cm^−1^. The bands at 781.12 cm^−1^, 626.82 cm^−1^, and 518.82 cm^−1^ are attributed to =C–H bending of benzene groups present in the polyphenol; indeed, OFI is an excellent source of phenolic antioxidants [41]. Bands at 1425.30 cm^−1^, 1319.22 cm^−1^, 1060.78 cm^−1^ are attributed to CH_2_ symmetric bending, C–H bending, the C–O bond of OH, and the C–O–C of the cellulose molecule, respectively. The band at 1251.72 cm^−1^ represents C–O stretching vibration [26]. Moreover, the bands between 1320 cm^−1^ and 1025 cm^−1^ are characteristic of C–N, P–OH and –COOH groups, indicating the presence of aromatic proteins, phosphoric groups, and polysaccharides [43]. The broad band at 1614.31 cm^−1^ is characteristic of C=O stretching vibration [21]. Bands at 2850.59 cm^−1^ and 2920.03 cm^−1^ are assigned to CH and –CH_2_ groups’ stretching vibration in cellulose chains [21]. Further, these bands confirm the presence of fibers [43]. Finally, the broad band ranging from around 3700–3000 cm^−1^, centered at 3415.70 cm^−1^, corresponds to the stretching vibration of the strong –OH and –NH groups in the acidic and aliphatic components of cellulose and aromatic proteins [21]. This result confirms that OFICP is a heterogeneous material composed of cellulose, carbohydrates, fibers, proteins, and phenolic components.

The agar spectrum showed a broad band ranging from around 3500–3000 cm^−1^, centered at 3415.70 cm^−1^, assigned to the stretching vibration of hydroxyl (O–H) groups. The band at 2923.88 cm^−1^ is attributed to CH stretching vibration. The band at around 1645.32 cm^−1^ is related to the bending vibration of H–O–H of crystallization water [59]. The band ranging from 1460.01 to 1373.22 cm^−1^ can be assigned to C-O–H bending vibration of –OH. The bands at 1078.13 cm^−1^ and 931.55 cm^−1^ are characteristic of the C–O stretching group of 3,6-anhydro-L-galactose with the contribution of the CC–O out-of-phase stretching of primary and secondary alcohols of saccharide rings [59]. The band at 892.98 cm^−1^ reflects C–H stretching of β-galactose [38]. The bands at 779.19 cm^−1^ and 590.18 cm^−1^ represent the skeletal bending of pyranose rings [38,59].

XG exhibits a similar spectrum to agar due to the similarity of their polysaccharide chemical structure (Figure 6). The broad band centered at 3417.63 cm^−1^ is attributed to the axial deformation of the OH group; the band at 2925.81 cm^−1^ represents the valence vibration of C–H; the band at 1731.96 cm^−1^ I characteristic of valence vibration of C=O ester, carboxylic acid, aldehydes, and ketones; the band at 1618.17 cm^−1^ can be assigned to the asymmetric strength of the carboxylate ion [60]; the bands at 1454.23 cm^−1^ and 1379.01 cm^−1^ are characteristic of the CH bending of the methyl groups; the band at 1272.93 cm^−1^ represents the C–O valence of alcohol; the band at 1064.63 cm^−1^ is attributed to COC of the ether function and the band at 1024.13 cm^−1^ may be attributed to the C–O function of glucose [38,56,60].

The GA spectrum displayed characteristic bands at 3382.91 cm^−1^, 2923.88 cm^−1^, 1627.81 cm^−1^, 1423.37 cm^−1^, 1072.35 cm^−1^, and 1033.77 cm^−1^, corresponding to the –OH, C-H bending, C=O stretching vibration, CH bending of the methyl groups, COC of the ether function, and C–O function of glucose, respectively [61]. As for the glycerol spectrum, it displays the following main characteristic bands; 3413.77 cm^−1^ of OH stretching; 2927.74 cm^−1^ and 2881.45 cm^−1^ attributed to the CH_2_ groups; 1643.24 cm^−1^ characteristic of water; bands at 1457.68 cm^−1^ and 1328.16 cm^−1^ assigned to the C–O–H bending; 1110.92 cm^−1^ representing C–C bending; 1041.49 cm^−1^ attributed to C–O–H bending; and bands at 995.20 cm^−1^, 925.77 cm^−1^, 856.34 cm^−1^ which are assigned to C–O–H bending in O–H [62].

The FTIR spectra of the prepared blend films, OFICP–agar, 3OFI CP-agar with different concentrations of glycerol, OFICP–agar-GA, and OFICP–agar-XG ternary blend films, provide information regarding the intermolecular interactions that occurred between film components, which are reflected by the bands’ position shifting [63,64].

The FTIR spectrum of OFICP–agar blend film without glycerol (F1-0%G) is presented in Figure 7. Merging, shifting, and disappearance of bands are the main changes observed, in comparison to the FTIR spectrum of pure OFICP and agar powder individually (Figure 6). In fact, the characteristic bands of OFICP and agar, 1614.31 cm^−1^ and 1645.32 cm^−1^, respectively, are merged into a single broad band at 1585.38 cm^−1^ with a shoulder at 1648.04 cm^−1^. The characteristic band of OH and NH groups of OFICP (3415.70 cm^−1^) and agar (3365.55 cm^−1^) are merged into a broad band ranging from 3556.49 to 3105.18 cm^−1^. Similarly, the bands at 2920.03 cm^−1^ and 2923.88 cm^−1^ of OFICP and agar, respectively, are merged into a single band at 2935.46 cm^−1^, and the bands at 1060.78 cm^−1^ (of OFICP) and 1078.13 cm^−1^ (of agar) are merged into a broad band at 1053.06 cm^−1^. Furthermore, the characteristic band at 1425.30 cm^−1^ of OFICP shifted to a lower wavenumber at 1415.65 cm^−1^; the bands at 1373.22 cm^−1^ and 1161.07 cm^−1^ of agar shifted to higher wavenumbers at 1384.79 cm^−1^, and 1166,83 cm^−1^, respectively, and were accompanied by an increase in their intensity. Meanwhile, the bands at 2850.59 cm^−1^ of OFICP and 1737.74 cm^−1^ of agar disappeared. These changes suggest strong intermolecular interactions between the functional groups of the OFICP and agar, thus indicating excellent miscibility [26,56,63,65]. It is well-established that the miscibility between film components is a crucial factor that affects the microstructure and the functional properties of blended films [63].

The effect of glycerol is evaluated by comparing the FTIR spectra of OFICP–agar blend film with different glycerol concentrations (F1-30%G, F1-40%G, F1-50%G) on the FTIR spectrum of OFICP–agar blend film without glycerol (F1-0%G, control), as shown in Figure 7.

When glycerol was added at 30% (film F1-30%G), a shift in the main bands to a lower or higher wavenumber with increased intensities and broadness was observed. The broadband at 3556.49–3105.18 cm^−1^ was improved with multiple peaks, increased in intensity and broadness, and slightly shifted to higher wavenumbers at 3573.85–3074.32 cm^−1^; the bands at 2935.46 cm^−1^ and 1053.06 cm^−1^ shifted to a lower wavenumber at 2925.81 cm^−1^ and 1049.20 cm^−1^, respectively. Meanwhile, the band at 1585.38 cm^−1^ shifted to a higher wavenumber at 1608.52 cm^−1^, with the disappearance of the shoulder observed at 1648.04 cm^−1^ in the FTIR spectra of the control film. In addition, the band at 1164.92 cm^−1^ appeared as a shoulder at 1157.21 cm^−1^. These results suggest the destruction of intermolecular hydrogen bonds between the OFICP and agar due to the insertion of the glycerol molecules and the formation of new hydrogen bonds between glycerol, OFICP, and agar, thus confirming that the glycerol was attracted and bound to the polymer matrix [42,56,57,64,65].

When the glycerol concentration increased to 40 and 50%, the most pronounced change observed was the merging of the band at 2925 cm^−1^ into the region ranging from 3560 to 2825 cm^−1^, thus increasing the band’s intensity and broadness, and the appearance of multiple new peaks within the domain ranging from 1450 cm^−1^ to 400 cm^−1^. This illustrates the formation of more new hydrogen bonds between glycerol, OFICP, and agar [57]. Furthermore, the characteristic bands of N-H and amide reappeared at 1589.23 cm^−1^ and 1666.38 cm^−1^, respectively. This suggests that higher concentrations of glycerol facilitate the expression of initial polymer functions due to increases in the intermolecular space between polymer chains [56,57,65]. The XRD results presented in the following section also support this suggestion.

GA addition to OFICP–agar-glycerol 30% blend film significantly affected the FTIR spectra and appeared to have changed the structure of the polymeric matrix, as shown in Figure 7. The characteristic bands of agar and GA at 1373.22 cm^−1^ and 1423.37 cm^−1^, respectively, appeared after the addition of 0.3 and 1.5 g of GA, respectively. This suggests the dissociation of intermolecular hydrogen bonds between the OFICP and agar by competition with GA and the insertion of GA into the film network [42]. In addition, new bands appeared at 1338.51 cm^−1^, 1365.51 cm^−1^, 1350.08 cm^−1^, and 1369.37cm^−1^, in parallel to the disappearance of characteristic bands of agar (854.41 cm^−1^, 892.98 cm^−1^, 925.77cm^−1^), after the addition of 0.5, 1, and 1.5 g of GA. Moreover, the main band at 1608.52 cm^−1^ (C=O bending groups) was gradually shifted to 1585.38 cm^−1^ and was accompanied by the appearance of a new peak at 1639.38 cm^−1^ after the addition of 0.3 g of GA; 1585.38 cm^−1^ was accompanied by two new peaks 1604.66 and 1662.52 cm^−1^ after the addition of 0.5 g of GA; 1573.81 cm^−1^ showed new peaks at 1604.66 and 1658.67 cm^−1^ in the film containing 1 g of GA; and 1589.23 cm^−1^ had a shoulder at 1654.74 cm^−1^ after the addition of 1.5 g of GA. Furthermore, the broadband ranging from 3105.18–3573.85 cm^−1^ gradually increased in intensity and broadness with increasing GA content. These changes suggest strong intermolecular interactions and crosslinking reactions between the GA and OFICP, thus indicating excellent miscibility [56].

On the other hand, adding XG to OFICP–agar-glycerol 30% blend film barely affected the FTIR spectra; however, the characteristic band of XG at 1623.75 cm^−1^ appeared when 0.5 g of XG was added, confirming its incorporation into the polymer network. Further, the band at 1608.51 cm^−1^ shifted to a lower wavenumber at 1593.09 cm^−1^ when 0.5 g of XG was added. The band at 1105.14 cm^−1^ shifted to a lower wavenumber at 1099.63 cm^−1^ when 0.3 XG was added and disappeared when 0.5 g of XG was added. These results agree with a similar report from [38] on blended films made of agar and XG gum. These authors observed a shifting of peaks to lower wavenumbers with increasing XG content, suggesting that this results from intermolecular interactions between the film components [38]. Comparing the effect of GA and XG addition on the FTIR spectrum of the OFICP–agar-glycerol 30% blend film, it appears that GA is more compatible with OFICP. The similarity of their chemical structure could justify this.

The strong intermolecular interactions and crosslinking reactions that occurred between the different film compounds increase the lifetime of the resulting blend films and preserve their performance [66,67].

### 3.6. X-ray Diffraction Analysis

X-ray diffraction analysis is one of the most methods used to characterize the structure and the miscibility of blend polymers. If the blended polymers are not miscible or have very-low compatibility, each polymer would have its own crystalline region in the blend films. Further, this method is used to determine the degree of crystallinity in polymers [68].

**Figure 8:**
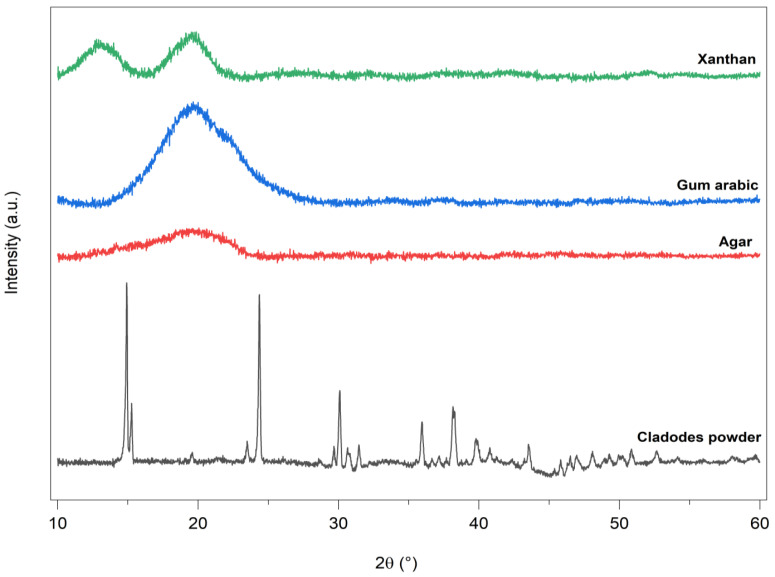
XRD patterns of OFICP, agar, GA, and XG.

**Figure 9:**
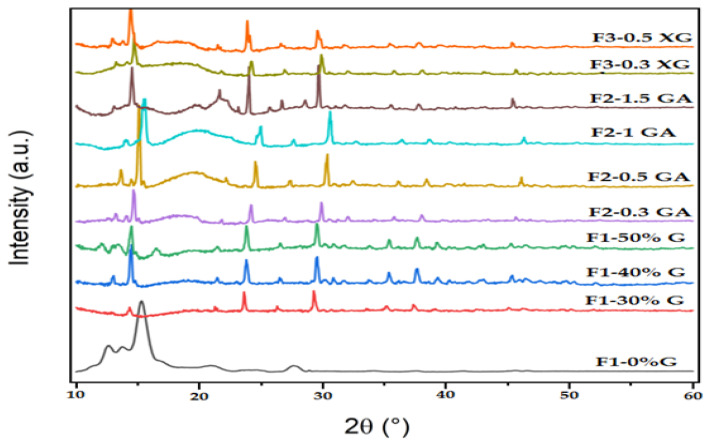
XRD patterns of OFICP–agar blend film with different concentrations of glycerol (F1-0%G–F1-50%G) and OFICP–agar-glycerol 30% blend film with varying contents of GA (F2-0.3GA–F2-1.5GA) and XG (F3-0.3XG–F3-0.5XG).

The XRD patterns of the starting materials (OFICP, agar, GA, and XG) and all the prepared blend films are presented in Figure 8 and Figure 9, respectively. The main peak positions are summarized in Table 3.

As shown in Figure 8, the XRD pattern of OFICP showed ten main peaks at 2θ = 14.9°, 15.2°, 23.49°, 24.3°, 29.67°, 30.09°, 35.9°, 38.15°, 39.78°, and 43.52°, which correspond to the chemical structure of calcium oxalate monohydrate Ca(C_2_O_4_)H_2_O) and cellulose I [21,43,69]. The 14.9°, 15.2°, and 24.3° peaks were attributed to cellulose I [21,43]. The crystallinity index of OFICP was estimated at 25%.

Agar, GA, and XG (Figure 8) showed similar XRD patterns with a broad amorphous peak centered at around 19.65°. This corresponds to the typical diffraction pattern of these gums, which have an amorphous structure in nature [37,39,43,56,70]. In addition, the XRD pattern of XG showed a second diffraction broad peak centered at 13.6°, and this may be due to the presence of a nanocrystalline phase which is also characterized by a broad diffraction halo, as indicated by Kushwaha et al. [71], Kumar et al. [72], and Fan et al. [36].

The addition of agar to OFICP to obtain OFICP–agar blend film (F1-0%G) gave rise to new peaks at 2θ = 12.7°, 13.7° and 27.62°, in addition to the main characteristic XRD peak of the OFICP, 15.2°, which shifted slightly to a higher diffraction angle at 15.33° with a significant increase in its intensity (Table 3, Figure 9). On the other hand, the other characteristic XRD peaks of OFICP at 14.92° did not appear in the OFICP–agar blend film XRD pattern, while the peaks from 29.67° to 43.52° became significantly less intense (Figure 9). Therefore, the crystallinity index of OFICP–agar blend film was estimated at 30%. Such results suggest the rearrangement of polymer chains and the formation of intermolecular interactions through hydrogen bonds between the chains of OFICP and agar in the blend film, which indicated excellent miscibility [29,73].

When glycerol was added at 30% (film F1-30%G), the main characteristic XRD peaks of the OFICP–agar blend film without glycerol (control), 12.7°, 13.74°, 15.32°, 24.45°, and 27.62°, disappeared, while the characteristic XRD peaks of the OFICP (starting material) reappeared with a slight shift to the left or right (Table 3). Meanwhile, the crystallinity index of the OFICP–agar-glycerol 30% blend film (F1-30%G) decreased to 6% (as compared with 30% of the OFICP–agar blend control film). This result may be due to glycerol action, which weakens the intermolecular forces between polymer chains and increases the interchain space, as mentioned previously [44,48,55,64,65].

However, when the glycerol concentration increased to 40 and 50% (film F1-40%G and F1-50%G), a right-shift in the XRD peaks’ position with an increase in their intensity was observed, thus resulting in the film’s crystallinity increasing to 9 and 8%, respectively (Table 3). Such a result may be due to the high concentrations of glycerol (40 and 50%), which increase the free volume between polymer chains, causing their enhanced molecular mobility and promoting molecular arrangement as well as the reappearance of the XRD peaks of the OFICP crystal compounds with an increase in their intensity, thus resulting in increases in the film’s crystallinity index [42,48,57,74].

The addition of 0.3, 0.5, and 1g of GA to OFICP–agar-glycerol 30% blend (F2-0.3GA, F2-0.5GA, and F2-1GA) caused a gradual right-shift in the XRD peaks’ position accompanied by a slight increase in their intensity, while an opposite behavior was observed at the highest content of GA 1.5 g (F2-1.5GA), which resulted in a left-shift in the XRD peaks’ position in addition to the appearance of new peaks at 25.67°, 26.66°, 28.58° (Figure 9, Table 3). Similarly, the XG addition at 0.3 and 0.5 g to OFICP–agar-glycerol 30% blend film (films F3-0.3XG and F3-0.5XG) resulted in a significant increase in the XRD peaks’ intensity and a left-shift in the peaks’ position. These results may be due to an increase in the interchain space caused by the presence of 30% glycerol, which is associated with an increased content of XG and GA [42,75]. Furthermore, the XRD peaks position shifting indicates an inter and intra-molecular hydrogen-bonding formation between the film compounds, thus reflecting excellent miscibility [38,42,65].

The presence of the crystalline components of OFICP in the different obtained blend film structures (reflected by the appearance of the characteristic XRD peaks of calcium oxalate in the films structure) confers the good stability and good barrier properties of the films by protecting them from light, thus increasing their lifetime [41].

### 3.7. Thermal Properties by Differential Scanning Calorimeter (DSC)

The glass transition temperature (T_g_) value and the heat capacity change (∆C p) obtained from the DSC thermogram for each prepared blend film are presented in Table 4. The T_g_ value determines the physical properties of polymers in relation to room temperature. The polymer remains soft and flexible if the Tg value is below room temperature. [76].

Our results revealed that all the prepared blend films exhibited a T_g_ below room temperature. Moreover, the T_g_ value and the ∆C p of the OFICP–agar blend film decreased with increasing glycerol concentration, as shown in Table 4. Indeed, a T_g_ value of −1.89 °C with ∆C p of 1.079 (µV) was observed for the OFICP–agar blend film without glycerol (F1-0%G, control). The addition of 40 and 50% glycerol to the OFICP–agar blend (F1-40%G, F1-50%G) significantly (*p* < 0.05) decreased the T_g_ values to −15.42 °C and −15.66 °C, respectively, with a ∆C p of 0.58 and 0.29 (µV), respectively. Similar T_g_ values were reported for many biopolymers, such as waxy maize starch film plasticized by sorbitol [77] and edible gum cordia film plasticized by polyethylene glycol 400 [57]. Chaudhary et al. [78] and Jouki et al. [48] found a similar profile for cress seed gum and starch plasticized by glycerol; the authors observed that increasing the glycerol concentration decreased the T_g_ value of films. The plasticization mechanism may explain this decrease in T_g_ value with the glycerol addition. In fact, plasticizers, such as glycerol reduce intermolecular forces between the polymer chains and consequently increase the interchain space and free volume in the polymer, resulting in a T_g_ value decrease [48,57,73]. Moreover, this decrease in the T_g_ value may be partly due to the higher moisture content in these films (as noted in the moisture content results section) as well as the the low Tg (−90 °C) of glycerol [57].

Similarly, the T_g_ value of the OFICP–agar-GA ternary blend film decreased with increasing GA content. Indeed, T_g_ values of −8.87 °C, −11.44 °C, −15.29 °C, and −31.31 °C were observed for the ternary blend films F2-0.3GA, F2-0.5GA, F2-1GA, and F2-1.5GA, respectively, with a ∆C p of 1.019, 0.174, −0.013, and −1.079 (µV), respectively. This decrease in the T_g_ value may be due to the structure of GA, characterized by a high degree of branching, which increases the intermolecular space between polymer chains and consequently decreases the T_g_ value [37,40,79].

On the other hand, the T_g_ value of the OFICP–agar-XG ternary blend film increased with increasing XG content. Indeed, the T_g_ value increased from −27.70 °C to −16.381 °C for the blend films with 0.3 g XG (F3-0.3XG) and 0.5 g XG (F3-0.5XG), respectively, with a ∆C p of 0.93 and 0.53 (µV), respectively. This result may be partly due to the ability of XG to create strong interactions with biopolymer chains and partly due to the higher molecular weight of XG resulting in increases in the T_g_ value of the ternary blend film [38,49,56,75].

**Figure 10:**
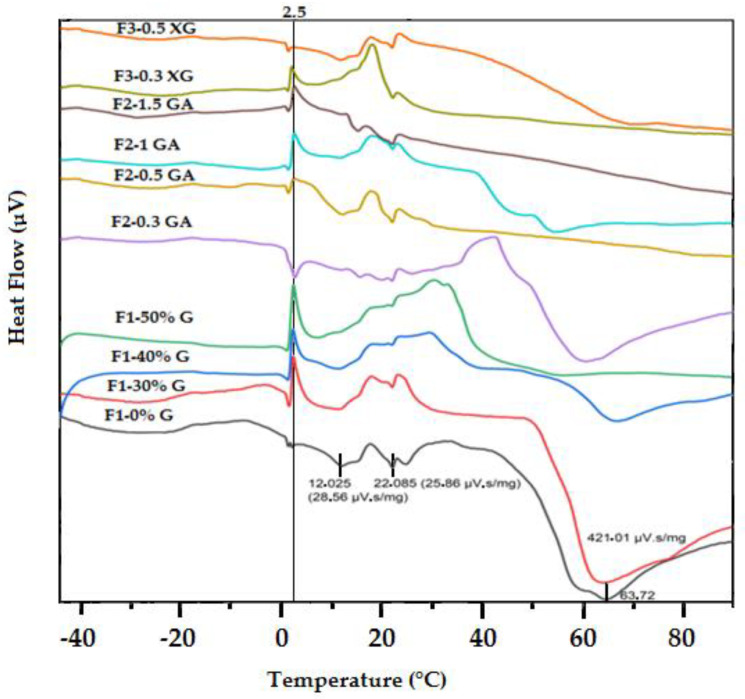
DSC thermograms of OFICP–agar blend film with different concentrations of glycerol (F1-0%G–F1-50%G) and OFICP–agar-glycerol 30% blend film with varying contents of GA (F2-0.3GA–F2-1.5GA) and XG (F3-0.3XG–F3-0.5XG).

DSC thermograms of all the prepared blend films displayed several peaks (Figure 10). The DSC thermogram for OFICP–agar blend film without glycerol (F1-0%G) showed three peaks, the first with an onset temperature of 7.48 °C and a peak at 12.025 °C at a heat of 28.56 (µV.s/mg), followed by a second peak with an onset temperature of 19.79 °C and a peak at 22.085 °C at the heat of 25.86 (µV.s/mg), and a third peak with an onset temperature of 50.75 °C and a peak at 63.72 °C at a heat of 421.01 (µV.s/mg).

The addition of glycerol, GA, and XG barely affected the DSC thermograms; however, new peak at temperatures ranging from 1.170 °C to 2.50 °C when the heat ranged from 1 to 50 (µV·s/mg) appeared. Furthermore, a decrease in the heat at the peak ranging from 50 to 60 °C with increasing glycerol concentration and GA content was observed. In fact, the addition of glycerol decreased the heat from 421.01 (µV·s/mg) for the blend film without glycerol (F1-0%G) to 347.836 and 139 (µV·s/mg) for the blend film with 30 and 40% glycerol (F1-30%G, F1-40%G), respectively, for the peaks at 62.91 and 66.33 °C, respectively. Similarly, the addition of GA decreased the heat from 251.50 (µV·s/mg) for the ternary blend film with 0.3 g GA (F2-0.3GA) to 23.13 (µV·s/mg) for the ternary blend film with 1 g GA (F2-1 GA) for the peaks at 58.56 and 52.86 °C, respectively. Such results may be attributed to the glycerol and GA impact, as explained above, in addition to the heterogeneity of our samples.

Similar thermal behavior was found for many other biopolymers, such as cactus mucilage-based films, agar-chitosan, and starch-glycerol. The authors suggested that this behavior is related to water vaporization and successive thermal events [57,76,80,81].

## 4. Conclusions

In the present study, OFICP–agar binary blend film, OFICP–agar-gum arabic, and OFICP–agar-xanthan ternary blend films were successfully eco-friendly prepared.

The results suggest that the OFICP has excellent film-forming properties when blended with gums agar, gum arabic, or xanthan; thus, it can be successfully used for flexible food packaging applications. FTIR and XRD analysis confirmed strong intermolecular interactions among the different blend film compounds, which enhance the blend films’ stability and physical, barrier, and thermal properties.

Due to OFICP’s richness in bioactive compounds, OFICP–agar, OFICP–agar-gum arabic, and OFICP–agar-xanthan blend films may serve as promising candidates for antimicrobial/antioxidant food packaging materials, for which an exhaustive determination of other film properties (mechanical and other barrier properties) is required.

## Figures and Tables

**Table 1:** Composition of the different film formulations for 100 mL film-forming suspensions.

Formulation Number	Codification	Ratio OFICP:A:GA:XG	OFICP (g)	Agar (A) (g)	Gum Arabic (GA) (g)	Xanthan (XG) (g)	Glycerol (G) (%)
1st formulation (F1)	F1-0%G	2:2:0:0	2	2	0	0	0
	F1-30%G	2:2:0:0	2	2	0	0	30
	F1-40%G	2:2:0:0	2	2	0	0	40
	F1-50%G	2:2:0:0	2	2	0	0	50
2nd formulation (F2)	F2-0.3GA	2:1.7: 0.3:0	2	1.7	0.3	0	30
	F2-0.5GA	2:1.5: 0.5:0	2	1.5	0.5	0	30
	F2-1GA	2:1:1:0	2	1	1	0	30
	F2-1.5GA	2:0.5:1.5:0	2	0.5	1.5	0	30
3rd formulation (F3)	F3-0.3XG	2:1.7:0:0.3	2	1.7	0	0.3	30
	F3-0.5XG	2:1.5:0:0.5	2	1.5	0	0.5	30
	F3-1XG	2:1:0:1	2	1	0	1	30

OFICP: *Opuntia ficus indica* cladodes powder; A: agar; G: glycerol; XG: xanthan; GA: gum arabic.

**Table 2:** Thicknesses of the prepared films at different formulations.

Formulation Number	Samples Code	Thickness (mm)
1st formulation (F1)	F1-0%G	0.056 ± 0.013 ^c^
	F1-30%G	0.069 ± 0.016 ^b^
	F1-40%G	0.084 ± 0.009 ^a^
	F1-50%G	0.095 ± 0.023 ^a^
2nd formulation (F2)	F2-0.3GA	0.123 ± 0.058 ^a^
	F2-0.5GA	0.124 ± 0.048 ^a^
	F2-1GA	0.098 ± 0.029 ^a,b^
	F2-1.5GA	0.090 ± 0.016 ^b^
3rd formulation (F3)	F3-0.3XG	0.105 ± 0.03 ^a^
	F3-0.5XG	0.101 ± 0.02 ^a^

Film formulations: F1-0%G (OFICP–agar); F1-30%G (OFICP–agar-glycerol 30%); F1-40%G (OFICP–agar-glycerol 40%); F1-50%G (OFICP–agar-glycerol 50%); F2-0.3GA (OFICP–agar-glycerol 30%-GA 0.3 g); F2-0.5GA (OFICP–agar-glycerol 30%-GA 0.5 g); F2-1GA (OFICP–agar-glycerol 30%-GA 1 g); F2-1.5GA (OFICP–agar-glycerol 30%-GA 1.5 g); F3-0.3XG (OFICP–agar-glycerol 30%-XG 0.3g); F3-0.5XG (OFICP–agar-glycerol 30%-XG 0.5 g). Thickness results expressed as means ± standard deviation (SD). Mean values in the same column with different superscript letters (^a-b-c^) are significantly different (*p* < 0.05).

**Table 3:** The position of main XRD peaks and crystallinity of OFICP and the prepared blend films.

Sample	Peak Position (°)													Crystallinity (%)
OFICP			14.92	15.27	23.49	24.37					29.67	35.95	38.15	25.89
F1-0%G	12.70	13.74		15.33	23.49	24.45			27.63					30.14
F1-30%G			14.34		23.62						29.26	35.19	37.35	6.20
F1-40%G			14.49		23.80						29.55	35.35	37.66	9.63
F1-50%G			14.49		23.80						29.52	35.37	37.66	8.64
F2-0.3GA			14.65			24.19					29.89	35.79	38.02	3.72
F2-0.5GA		13.63		15.12		24.53					30.38		38.41	4.64
F2-1GA			14.83	15.61		24.99					30.59		38.67	4.49
F2-1.5GA			14.55			24.01	25.67	26.66		28.59	29.70			7.60
F3-0.3XG			14.70			24.21					29.89	35.76	38.13	5.051
F3-0.5XG			14.44		23.88	24.09					29.57	35.42	37.79	5.033

Film formulations: F1-0%G (OFICP–agar); F1-30%G (OFICP–agar-glycerol 30%); F1-40%G (OFICP–agar-glycerol 40%); F1-50%G (OFICP–agar-glycerol 50%); F2-0.3GA (OFICP–agar-glycerol 30%-GA 0.3 g); F2-0.5GA (OFICP–agar-glycerol 30%-GA 0.5 g); F2-1GA (OFICP–agar-glycerol 30%-GA 1 g); F2-1.5GA (OFICP–agar-glycerol 30%-GA 1.5 g); F3-0.3XG (OFICP–agar-glycerol 30%-XG 0.3 g); F3-0.5XG (OFICP–agar-glycerol 30%-XG 0.5 g).

**Table 4:** T_g_ values and ∆C p of the prepared films at different formulations.

Samples Code	Glass Transition (°C)/ ∆C p (µV)			
	T_ig_	T_g_ (Peak)	T_eg_	∆C p (µV)
F1-0%G	03.59	01.89	0.966	1.079
F1-30%G	ND	ND	ND	ND
F1-40%G	16.45	15.42	15.073	0.576
F1-50%G	16,80	15.67	16.246	0.29
F2-0.3GA	11.36	08.87	9.633	1.019
F2-0.5GA	11.62	11.45	11.018	0.174
F2-1GA	15.98	15.29	16.423	0.013
F2-1.5GA	23.22	31.31	31.175	1.079
F3-0.3XG	27.60	27.70	25.573	0.932
F3-0.5XG	16.981	16.381	15.34	0.53

Film formulations: F1-0%G (OFICP–agar); F1-30%G (OFICP–agar-glycerol 30%); F1-40%G (OFICP–agar-glycerol 40%); F1-50%G (OFICP–agar-glycerol 50%); F2-0.3GA (OFICP–agar-glycerol 30%-GA 0.3 g); F2-0.5GA (OFICP–agar-glycerol 30%-GA 0.5 g); F2-1GA (OFICP–agar-glycerol 30%-GA 1 g); F2-1.5GA (OFICP–agar-glycerol 30%-GA 1.5 g); F3-0.3XG (OFICP–agar-glycerol 30%-XG 0.3g); F3-0.5XG (OFICP–agar-glycerol 30%-XG 0.5 g). T_ig_: Initiation temperature, T_eg_: final temperature. ND: Not detected.

## Data Availability

Data is contained within the article.

## References

[1] Han J.W., Ruiz-Garcia L., Qian J.P., Yang X.T. (2018). Food Packaging: A Comprehensive Review and Future Trends. Compr. Rev. Food Sci. Food Saf..

[2] Zare M., Namratha K., Ilyas S., Sultana A., Hezam A., Sunil L., Surmeneva M.A., Surmenev R.A., Nayan M.B., Ramakrishna S. (2022). Emerging Trends for ZnO Nanoparticles and Their Applications in Food Packaging. ACS Food Sci. Technol..

[3] Chen X., Ma W., Hu W., Liu Z., Wang H., Chen Y., Li L. (2023). Progress in release-activated food packaging films. Packag. Technol. Sci..

[4] Groh K.J., Backhaus T., Carney-Almroth B., Geueke B., Inostroza P.A., Lennquist A., Leslie H.A., Maffini M., Slunge D., Trasande L. (2019). Overview of known plastic packaging-associated chemicals and their hazards. Sci. Total Environ..

[5] Kan M., Miller S.A. (2022). Environmental impacts of plastic packaging of food products. Resour. Conserv. Recycl..

[6] Real L.E.P. (2023). Plastics Statistics: Production, Recycling, and Market Data. Recycled Materials for Construction Applications.

[7] Oudir M., Chader H., Bouzid B., Bendisari K., Latreche B., Boudalia S., Iguer-Ouada M. (2018). Male rat exposure to low dose of di (2-ethylhexyl) phthalate during pre-pubertal, pubertal and post-pubertal periods: Impact on sperm count, gonad histology and testosterone secretion. Reprod. Toxicol..

[8] Rudel R.A., Gray J.M., Engel C.L., Rawsthorne T.W., Dodson R.E., Ackerman J.M., Rizzo J., Nudelman J.L., Brody J.G. (2011). Food packaging and bisphenol A and bis(2-ethyhexyl) phthalate exposure: Findings from a dietary intervention. Environ. Health Perspect..

[9] Nerín C., Tovar L., Djenane D., Camo J., Salafranca J., Beltrán J.A., Roncalés P. (2006). Development of a new antioxidant active packaging for fresh meat. J. Agric. Food Chem..

[10] Djenane D., Beltrán J.A., Camo J., Roncalés P. (2016). Influence of vacuum at different ageing times and subsequent retail display on shelf life of beef cuts packaged with active film under high O_2_. J. Food Sci. Technol..

[11] Lammi S., Le Moigne N., Djenane D., Gontard N., Angellier Coussy H. (2018). Dry fractionation of olive pomace for the development of food packaging biocomposites. Ind. Crops Prod..

[12] Ahmed W., Azmat R., Khojah E., Ahmed R., Qayyum A., Shah A.N., Abbas A., Moin S., Samra B.N. (2022). The development of a green innovative bioactive film for industrial application as a new emerging technology to protect the quality of fruits. Molecules.

[13] Narasagoudr S.S., Masti S.P., Hegde V.G., Chougale R.B. (2023). Cetrimide crosslinked chitosan/guar gum/gum ghatti active biobased films for food packaging applications. J. Polym. Environ..

[14] Ait Ouahioune L., Wrona M., Becerril R., Salafranca J., Nerín C., Djenane D. (2022). *Ceratonia siliqua* L. kibbles, seeds and leaves as a source of volatile bioactive compounds for antioxidant food biopackaging applications. Food Packag. Shelf Life.

[15] Ratna, Ulfariati C., Yusmanizar, Aprilia S., Rahmiati, Munawar A.A. (2023). Development of biocomposite edible film food packaging based on gelatin from chicken claw waste. Case Stud. Chem. Environ. Eng..

[16] Gürler N., Torğut G. (2022). Physicomechanical, thermal and dielectric properties of eco-friendly starch-microcrystalline cellulose-clay nanocomposite films for food packaging and electrical applications. Packag. Technol. Sci..

[17] Freitas E.E.S., Dias Ê.R., Albuquerque M.M.S., Biondi I.B., Branco C.R.C., Cruz R.S., Branco A., Camilloto G.P. (2024). Antioxidant films based on poly(lactic acid) incorporated with crude extract from *Malpighia emarginata* DC pomace for use in food packaging. Packag. Technol. Sci..

[18] Martins M., Ribeiro M.H., Almeida C.M.M. (2023). Physicochemical, nutritional, and medicinal properties of *Opuntia ficus-indica* (L.) Mill. and its main agro-industrial use: A review. Plants.

[19] Dick M., Limberger C., Thys R.C.S., de Oliveira Rios A., Flôres S.H. (2020). Mucilage and cladode flour from cactus (*Opuntia monacantha*) as alternative ingredients in gluten-free crackers. Food Chem..

[20] FAOSTAT (2022). Food and Agriculture Organization. Élaboration D’une Stratégie de Développement de la Filière du Figuier de Barbarie (*Opuntia ficus-indica* L.) en Algérie—TCP/ALG/3702. www.fao.org.

[21] Maaoui A., Trabelsi A.B.H., Hamdi M., Chagtmi R., Jamaaoui F., Lopez G., Cortazar M., Olazar M. (2023). Towards local circular economy through *Opuntia Ficus-indica* cladodes conversion into renewable biofuels and biochars: Product distribution and kinetic modelling. Fuel.

[22] Hernández-Becerra E., de los Angeles Aguilera-Barreiro M., Contreras-Padilla M., Pérez-Torrero E., Rodriguez-Garcia M.E. (2022). Nopal cladodes (*Opuntia ficus indica*): Nutritional properties and functional potential. J. Funct. Foods.

[23] De Andrade Vieira É., de Magalhães Cordeiro A.M.T. (2022). Bioprospecting and potential of cactus mucilages: A bibliometric review. Food Chem..

[24] Di Lorenzo F., Silipo A., Molinaro A., Parrilli M., Schiraldi C., D’Agostino A., Izzo E., Rizza L., Bonina A., Bonina F. (2017). The polysaccharide and low molecular weight components of *Opuntia ficus indica* cladodes: Structure and skin repairing properties. Carbohydr. Polym..

[25] Espino-Díaz M., De Jesús Ornelas-Paz J., Martínez-Téllez M.A., Santillán C., Barbosa-Cánovas G.V., Zamudio-Flores P.B., Olivas G.I. (2010). Development and characterization of edible films based on mucilage of *Opuntia ficus-indica* (L.). J. Food Sci..

[26] Gheribi R., Puchot L., Verge P., Jaoued-Grayaa N., Mezni M., Habibi Y., Khwaldia K. (2018). Development of plasticized edible films from *Opuntia ficus*-*indica* mucilage: A comparative study of various polyol plasticizers. Carbohydr. Polym..

[27] Olawuyi I.F., Kim S.R., Lee W.Y. (2021). Application of plant mucilage polysaccharides and their techno-functional properties’ modification for fresh produce preservation. Carbohydr. Polym..

[28] Gheribi R., Khwaldia K. (2019). Cactus mucilage for food packaging applications. Coatings.

[29] Makhloufi N., Chougui N., Rezgui F., Benramdane E., Silvestre A.J., Freire C.S., Vilela C. (2022). Polysaccharide-based films of cactus mucilage and agar with antioxidant properties for active food packaging. Polym. Bull..

[30] Barba F.J., Garcia C., Fessard A., Munekata P.E., Lorenzo J.M., Aboudia A., Ouadia A., Remize F. (2022). *Opuntia ficus indica* edible parts: A food and nutritional security perspective. Food Rev. Int..

[31] Msaddak L., Abdelhedi O., Kridene A., Rateb M., Belbahri L., Ammar E., Nasri M., Zouari N. (2017). *Opuntia ficus-indica* cladodes as a functional ingredient: Bioactive compounds profile and their effect on antioxidant quality of bread. Lipids Health Dis..

[32] Makhloufi N., Chougui N., Rezgui F., Benramdane E., Freire C.S., Vilela C., Silvestre A.J. (2021). Bio-based sustainable films from the Algerian *Opuntia ficus*-*indica* cladodes powder: Effect of plasticizer content. J. Appl. Polym. Sci..

[33] Scaffaro R., Maio A., Gulino E.F., Megna B. (2019). Structure-property relationship of PLA-*Opuntia Ficus-indica* biocomposites. Compos. Part B Eng..

[34] Wang L.-F., Rhim J.-W. (2015). Preparation and application of agar/alginate/collagen ternary blend functional food packaging films. Int. J. Biol. Macromol..

[35] Chacon W.D.C., Paz-Arteaga S.L., Torres-León C., Valencia G.A. (2023). Gums-Based Coatings Applied to Extend the Shelf Life of Foods: A Review. J. Polym. Environ..

[36] Fan Y., Yang J., Duan A., Li X. (2021). Pectin/sodium alginate/xanthan gum edible composite films as the fresh-cut package. Int. J. Biol. Macromol..

[37] Kang S., Xiao Y., Guo X., Huang A., Xu H. (2021). Development of gum arabic-based nanocomposite films reinforced with cellulose nanocrystals for strawberry preservation. Food Chem..

[38] Rukmanikrishnan B., Rajasekharan S.K., Lee J., Lee J. (2019). Biocompatible agar/xanthan gum composite films: Thermal, mechanical, UV, and water barrier properties. Polym. Adv. Technol..

[39] Tahsiri Z., Mirzaei H., Hosseini S.M.H., Khalesi M. (2019). Gum arabic improves the mechanical properties of wild almond protein film. Carbohydr. Polym..

[40] Zibaei R., Hasanvand S., Hashami Z., Roshandel Z., Rouhi M., de Toledo Guimarães J., Mortazavian A.M., Sarlak Z., Mohammadi R. (2021). Applications of emerging botanical hydrocolloids for edible films: A review. Carbohydr. Polym..

[41] De Farias P.M., de Vasconcelos L.B., da Silva Ferreira M.E., Alves Filho E.G., De Freitas V.A., Tapia-Blácido D.R. (2021). Nopal cladode as a novel reinforcing and antioxidant agent for starch-based films: A comparison with lignin and propolis extract. Int. J. Biol. Macromol..

[42] Yoksan R., Dang K.M. (2023). The effect of polyethylene glycol sorbitan monostearate on the morphological characteristics and performance of thermoplastic starch/biodegradable polyester blend films. Int. J. Biol. Macromol..

[43] De Farias P.M., de Vasconcelos L.B., Ferreira M.E., Pascall M., Tapia-Blácido D.R. (2021). Nopal cladode (*Opuntia ficus-indica*) flour: Production, characterization, and evaluation for producing bioactive film. Food Packag. Shelf Life.

[44] Priyadarshi R., Kumar B., Negi Y.S. (2018). Chitosan film incorporated with citric acid and glycerol as an active packaging material for extension of green chilli shelf life. Carbohydr. Polym..

[45] Djenane D., Ben Miri Y., Ariño A. (2023). Use of algerian type *Ras El-Hanout* spices mixture with marination to increase the sensorial quality, shelf life, and safety of whole rabbit carcasses under low-O_2_ modified atmosphere packaging. Foods.

[46] Ait Ouahioune L., Wrona M., Nerín C., Djenane D. (2022). Novel active biopackaging incorporated with macerate of carob (*Ceratonia siliqua* L.) to extend shelf-life of stored Atlantic salmon fillets (*Salmo salar* L.). LWT Food Sci. Technol..

[47] De Carli C., Aylanc V., Mouffok K.M., Santamaria-Echart A., Barreiro F., Tomás A., Pereira C., Rodrigues P., Vilas-Boas M., Falcão S.I. (2022). Production of chitosan-based biodegradable active films using bio-waste enriched with polyphenol propolis extract envisaging food packaging applications. Int. J. Biol. Macromol..

[48] Jouki M., Khazaei N., Ghasemlou M., HadiNezhad M. (2013). Effect of glycerol concentration on edible film production from cress seed carbohydrate gum. Carbohydr. Polym..

[49] Kim S.R.B., Choi Y.-G., Kim J.-Y., Lim S.-T. (2015). Improvement of water solubility and humidity stability of tapioca starch film by incorporating various gums. LWT Food Sci. Technol..

[50] Ratna, Aprilia S., Arahman N., Bilad M.R., Suhaimi H., Munawar A.A., Nasution I.S. (2022). Bio-nanocomposite based on edible gelatin film as active packaging from *Clarias gariepinus* fish skin with the addition of cellulose nanocrystalline and nanopropolis. Polymers.

[51] Kaya M., Ravikumar P., Ilk S., Mujtaba M., Akyuz L., Labidi J., Salaberria A.M., Cakmak Y.S., Erkul S.K. (2018). Production and characterization of chitosan based edible films from Berberis crataegina’s fruit extract and seed oil. Innov. Food Sci. Emerg. Technol..

[52] Khodaei D., Oltrogge K., Hamidi-Esfahani Z. (2020). Preparation and characterization of blended edible films manufactured using gelatin, tragacanth gum and, Persian gum. LWT Food Sci. Technol..

[53] Tessaro L., Lourenço R.V., Martelli-Tosi M., do Amaral Sobral P.J. (2021). Gelatin/chitosan based films loaded with nanocellulose from soybean straw and activated with “Pitanga” (*Eugenia uniflora* L.) leaf hydroethanolic extract in W/O/W emulsion. Int. J. Biol. Macromol..

[54] Kim S., Ustunol Z. (2001). Solubility and moisture sorption isotherms of whey-protein-based edible films as influenced by lipid and plasticizer incorporation. J. Agric. Food Chem..

[55] Arham R., Salengke S., Metusalach M., Mulyati M. (2018). Optimization of agar and glycerol concentration in the manufacture of edible film. Int. Food Res. J..

[56] Hazirah M.N., Isa M., Sarbon N. (2016). Effect of xanthan gum on the physical and mechanical properties of gelatin-carboxymethyl cellulose film blends. Food Packag. Shelf Life.

[57] Haq M.A., Hasnain A., Azam M. (2014). Characterization of edible gum cordia film: Effects of plasticizers. LWT Food Sci. Technol..

[58] Arismendi C., Chillo S., Conte A., Del Nobile M.A., Flores S., Gerschenson L.N. (2013). Optimization of physical properties of xanthan gum/tapioca starch edible matrices containing potassium sorbate and evaluation of its antimicrobial effectiveness. LWT Food Sci. Technol..

[59] Bertasa M., Botteon A., Brambilla L., Riedo C., Chiantore O., Poli T., Sansonetti A., Scalarone D. (2017). Cleaning materials: A compositional multi-analytical characterization of commercial agar powders. J. Anal. Appl. Pyrolysis.

[60] Naji-Tabasi S., Shahidi-Noghabi M., Dovom A.M. (2023). Investigating the fabrication and functional properties of new composite hydrogels containing gellan/alginate/xanthan gum. J. Sol-Gel Sci. Technol..

[61] Karaaslan M., Şengün F., Cansu Ü., Başyiğit B., Sağlam H., Karaaslan A. (2021). Gum arabic/maltodextrin microencapsulation confers peroxidation stability and antimicrobial ability to pepper seed oil. Food Chem..

[62] Bokovets S.P., Pertsevoi F.V., Murlykina N.V., Smetanska I.M., Borankulova A.S., Ianchyk M.V., Omelchenko S.B., Grinchenko O.O., Grychenko N.G., Dikhtyar A.M. (2023). Investigation of infrared spectra of agar-based gel systems for the production of jelly bars. J. Chem. Technol..

[63] Wanchoo R., Sharma P. (2003). Viscometric study on the compatibility of some water-soluble polymer–polymer mixtures. Eur. Polym. J..

[64] Jaderi Z., Tabatabaee Yazdi F., Mortazavi S.A., Koocheki A. (2023). Effects of glycerol and sorbitol on a novel biodegradable edible film based on Malva sylvestris flower gum. Food Sci. Nutr..

[65] Liu H., Adhikari R., Guo Q., Adhikari B. (2013). Preparation and characterization of glycerol plasticized (high-amylose) starch–chitosan films. J. Food Eng..

[66] Laycock B., Nikolić M., Colwell J.M., Gauthier E., Halley P., Bottle S., George G. (2017). Lifetime prediction of biodegradable polymers. Prog. Polym. Sci..

[67] Plota A., Masek A. (2020). Lifetime prediction methods for degradable polymeric materials—A short review. Materials.

[68] Kurt A., Toker O.S., Tornuk F. (2017). Effect of xanthan and locust bean gum synergistic interaction on characteristics of biodegradable edible film. Int. J. Biol. Macromol..

[69] Contreras-Padilla M., Rivera-Muñoz E.M., Gutiérrez-Cortez E., Del López A.R., Rodríguez-García M.E. (2015). Characterization of crystalline structures in *Opuntia ficus-indica*. J. Biol. Phys..

[70] Zheng M., Chen J., Tan K.B., Chen M., Zhu Y. (2022). Development of hydroxypropyl methylcellulose film with xanthan gum and its application as an excellent food packaging bio-material in enhancing the shelf life of banana. Food Chem..

[71] Kushwaha A.K., John M., Misra M., Menezes P.L. (2021). Nanocrystalline Materials: Synthesis, Characterization, Properties, and Applications. Crystals.

[72] Kumar A., Kumar R., Bijalwan P., Dutta M., Banerjee A., Laha T. (2019). Fe-based amorphous/nanocrystalline composite coating by plasma spraying: Effect of heat input on morphology, phase evolution and mechanical properties. J. Alloys Compd..

[73] Mostafavi F.S., Zaeim D. (2020). Agar-based edible films for food packaging applications—A review. Int. J. Biol. Macromol..

[74] de Morais Lima M., Carneiro L.C., Bianchini D., Dias A.R.G., Zavareze E.d.R., Prentice C., Moreira A.d.S. (2017). Structural, thermal, physical, mechanical, and barrier properties of chitosan films with the addition of xanthan gum. J. Food Sci..

[75] Sun Y., Ding R., Hong S.Y., Lee J., Seo Y.-K., Nam J.-D., Suhr J. (2021). MXene-xanthan nanocomposite films with layered microstructure for electromagnetic interference shielding and Joule heating. Chem. Eng. J..

[76] Otálora M.C., Carriazo J.G., Iturriaga L., Nazareno M.A., Osorio C. (2015). Microencapsulation of betalains obtained from cactus fruit (*Opuntia ficus-indica*) by spray drying using cactus cladode mucilage and maltodextrin as encapsulating agents. Food Chem..

[77] Viguié J., Molina-Boisseau S., Dufresne A. (2007). Processing and characterization of waxy maize starch films plasticized by sorbitol and reinforced with starch nanocrystals. Macromol. Biosci..

[78] Chaudhary D.S., Adhikari B.P., Kasapis S. (2011). Glass-transition behaviour of plasticized starch biopolymer system—A modified Gordon–Taylor approach. Food Hydrocoll..

[79] Aphibanthammakit C., Nigen M., Gaucel S., Sanchez C., Chalier P. (2018). Surface properties of Acacia senegal vs Acacia seyal films and impact on specific functionalities. Food Hydrocoll..

[80] Manhivi V.E., Venter S., Amonsou E.O., Kudanga T. (2018). Composition, thermal and rheological properties of polysaccharides from amadumbe (*Colocasia esculenta*) and cactus (*Opuntia* spp.). Carbohydr. Polym..

[81] Rivera-Corona J.L., Rodríguez-González F., Rendón-Villalobos R., García-Hernández E., Solorza-Feria J. (2014). Thermal, structural and rheological properties of sorghum starch with cactus mucilage addition. LWT Food Sci. Technol..

